# Functional Synergy between Cholecystokinin Receptors CCKAR and CCKBR in Mammalian Brain Development

**DOI:** 10.1371/journal.pone.0124295

**Published:** 2015-04-15

**Authors:** Sayoko Nishimura, Kaya Bilgüvar, Keiko Ishigame, Nenad Sestan, Murat Günel, Angeliki Louvi

**Affiliations:** 1 Department of Neurosurgery, Yale School of Medicine, New Haven, Connecticut, United States of America; 2 Yale Program on Neurogenetics, Yale School of Medicine, New Haven, Connecticut, United States of America; 3 Department of Genetics, Yale School of Medicine, New Haven, Connecticut, United States of America; 4 Yale Center for Genome Analysis, Yale School of Medicine, New Haven, Connecticut, United States of America; 5 Department of Neurobiology, Yale School of Medicine, New Haven, Connecticut, United States of America; University of North Dakota, UNITED STATES

## Abstract

Cholecystokinin (CCK), a peptide hormone and one of the most abundant neuropeptides in vertebrate brain, mediates its actions via two G-protein coupled receptors, CCKAR and CCKBR, respectively active in peripheral organs and the central nervous system. Here, we demonstrate that the CCK receptors have a dynamic and largely reciprocal expression in embryonic and postnatal brain. Using compound homozygous mutant mice lacking the activity of both CCK receptors, we uncover their additive, functionally synergistic effects in brain development and demonstrate that CCK receptor loss leads to abnormalities of cortical development, including defects in the formation of the midline and corpus callosum, and cortical interneuron migration. Using comparative transcriptome analysis of embryonic neocortex, we define the molecular mechanisms underlying these defects. Thus we demonstrate a developmental, hitherto unappreciated, role of the two CCK receptors in mammalian neocortical development.

## Introduction

Neuropeptides modulate neuronal activity in the mammalian central nervous system (CNS) [1]. Cholecystokinin (CCK), originally discovered in the gastrointestinal tract [2], is one of the most abundant neuropeptides [3] and mediates its actions via two G-protein coupled receptors, CCKAR and CCKBR, which have distinct pharmacology and largely non-overlapping and species-dependent expression in different organs. CCK has emerged as a central regulator of neuronal circuits [4], and has been implicated together with its receptors in the neurobiology of feeding, memory, nociception and exploratory behavior [5, 6] and further associated with neuropsychiatric disorders [4, 7, 8].

CCKAR is the peripheral receptor, having limited expression in the brain [9, 10], whereas CCKBR predominates in the CNS, mostly in neocortical and limbic structures; in the periphery, it is restricted to the stomach, where it serves as a receptor for gastrin, a hormone homologous to CCK [9, 11, 12]. In brain, CCKAR and CCKBR have distinct distribution and selectivity in different rodent species, as suggested by binding assays of radiolabeled CCK peptides [13–18]. CCK is expressed in neocortical pyramidal neurons, including corticocortical projection neurons [19–22] and in a distinct subtype of interneurons [23–29].

Genetic inactivation of *Cck* [30, 31], *Cckar* [17] or *Cckbr* [32, 33] leads to defects in the gastrointestinal system, satiation and control of food intake, memory and exploration, and anxiety-related behaviors [17, 31–42]. CCKAR regulates the migration of gonadotrophin-releasing hormone 1 neurons and olfactory bulb interneurons, as well as female sexual behavior [43–46], suggesting a broader role despite its restricted expression in the brain. On the other hand, *Cckbr* and *Cck* mutant mice appear to lack remarkable brain phenotypes [30–33].

In this study, we show that mutant mice lacking both CCK receptors have abnormalities of cortical development, including defects in the formation of the corpus callosum and interneuron migration. Using comparative transcriptome analysis of embryonic neocortex we define the molecular mechanisms underlying these defects. We thus demonstrate a hitherto unappreciated, synergistic role of CCK receptors in mammalian neocortical development.

## Materials and Methods

### Transgenic mice

This study was performed in strict accordance with the recommendations in the Guide for the Care and Use of Laboratory Animals of the National Institutes of Health. The protocol was approved by the Institutional Animal Care and Use Committee of Yale University (protocol number 2013–10886). All experimental procedures involving animals were performed under deep anesthesia, and all efforts were made to minimize suffering. Approximately two hundred mice were used in this study. Mice were sacrificed by injectable anesthetic overdose followed by cervical dislocation. Mice of either sex were used throughout the study. Details regarding the generation and characterization of *Cckar/Cckbr* double homozygous mutant mice (JAX 129-*Cckar* ^*tm1Kpn*^ *Cckbr* ^*tm1Kpn*^/J; stock number 006365) were previously reported [17, 33]. Genotyping of these transgenic mice was performed by PCR (*Cckar* ^*tm1Kpn*^ mutant forward primer: 5’-GAC AAT CGG CTG CTC TGA TG-3’, WT forward primer: 5’-GCT GCA TAG CGT CAC TTG G-3’, WT reverse primer: 5’-GAT GGA GTT AGA CTG CAA CC-3’, *Cckbr* ^*tm1Kpn*^ mutant forward primer: 5’-CTT GGG TGG AGA GGC TAT TC-3’, WT forward primer: 5’-CCA AGC TGC TGG CTA AGA AG-3’, WT reverse primer: 5’-CTT AGC CTG GAC AGA GAA GC-3’; additional information regarding PCR programs can be obtained on request).

### Histological analysis

Brains were perfused with 4% paraformaldehyde (PFA) in 0.1 M phosphate buffer, post-fixed and cryoprotected in 30% sucrose in 4% PFA, then sectioned on a cryomicrotome (Leica Microsystems, Wetzlar, Germany). Nissl staining was performed by standard procedures on serial sections of the forebrain. The area of the SVZ and RMS, distinguishable because of high cell density, was measured using NIH ImageJ on Nissl-stained 36-μm-thick coronal sections.

### *In situ* hybridization

Embryonic and postnatal mouse brains were fixed, respectively, by immersion in or intracardial perfusion with 4% PFA, post-fixed in 30% sucrose in 4% PFA and sectioned on a cryomicrotome (Leica Microsystems, Wetzlar, Germany). Human tissue was obtained from several sources, including the Human Fetal Tissue Repository at the Albert Einstein College of Medicine (New York, NY). Serial representative sections along the anterior/posterior axis of the neocortex (generally every third for embryonic and early postnatal or every sixth for adult) were processed for in situ hybridization as described previously [31]. RNA probes complementary to mouse *Cckar* (IMAGE: 4236240; BC020534), *Ccckbr*, *Cck*, *claudin 11* (gift from S. Tsukita, Kyoto University, Kyoto, Japan), *Ctgf*, *Tle4*, *Er81*, *Rorb* (gifts from C. Ragsdale, University of Chicago, Chicago, IL), *Tag1* (gift from D. Karagogeos, IMBB, Heraklion, Greece), *Gad1*, *reelin*, *Lhx6*, *Cxcl12*, *Bmp7* (gifts from E. Grove, University of Chicago, Chicago, IL) and *Nrp2* or to human *CCKAR*, *CCKBR* and *CCK*, were labeled with digoxigenin-11-UTP following cDNA cloning (S1 Table). Sections were analyzed using a Stemi stereomicroscope or AxioImager (Zeiss, Oberkochen, Germany) fitted with an AxioCam MRc5 digital camera. Images were captured using AxioVision software (Zeiss) and assembled in Adobe Photoshop.

### Layer Distribution Analysis

To quantify the distribution of neurons, anatomically matched sections (The Mouse Brain Atlas [47]) were selected. The postnatal neocortex was divided radially into 5 equal-sized bins from the pia to the upper edge of the white matter. The cells in each bin were quantified and reported as the percentage of total cells counted.

### Quantitative Analysis

Data were analyzed by two-tailed Student’s *t*-tests with a significance level of at least P< 0.05 for all statistical comparisons. Numbers of replicates are given in the main text or Fig legends.

### Labeling of callosal projections using DiI

Embryonic or postnatal mouse brains were fixed, respectively, by immersion in or intracardial perfusion with 4% PFA. Labeling of callosal projection neurons was achieved by placing DiI crystals (Molecular Probes, Life Technologies) in the cingulate or somatosensory cortex. The brains were returned to 4% PFA, placed in light-tight containers, and incubated at 37^°^C or at room temperature for 3–5 weeks. The brains were then embedded in 3% agarose and sectioned using a vibratome (Leica Microsystems).

### RNA sequencing

Embryonic brains (E17.5) were dissected in ice-cold Hank’s balanced salt solution (HBSS) (without Ca^2+^ or Mg^2+^) supplemented with 0.5% D-glucose and 25mM HEPES, the meninges removed and the neocortex microdissected. RNA was isolated by Trizol and purified with the RNeasy kit (Qiagen, Valencia, CA). The quality of the RNA was evaluated by A260/A280 ratio, and by electrophoresis on an Agilent Bioanalyzer (Agilent Technologies, Palo Alto, CA). RNA was amplified using the Ovation RNA-Seq system (NuGEN, San Carlos, CA) prior to library preparation. The sequencing library was prepared using the mRNA Seq Kit supplied by Illumina (San Diego, CA) according to the manufacturer’s protocol. Briefly, mRNA was isolated from total RNA using oligo dT on magnetic beads. The mRNA was then fragmented at 94^°^C and converted into double stranded cDNA. Following polishing of the cDNA ends and addition of adenine bases at the 3’ ends, specific adaptors supplied by Illumina were ligated. The adaptor ligated DNA was amplified by 15 cycles of PCR. The amplified DNA was then purified on the Qiagen PCR purification kit. The insert size and DNA concentration of the sequencing library were determined on an Agilent Bioanalyzer. Following cluster generation via isothermal solid support bridge amplification, one lane of single-read sequencing was performed for each experiment at a read length of 74 base pairs on the Genome Analyzer IIx (Illumina) according to the manufacturer’s protocol. Image analysis and base calling were performed by the Illumina pipeline version 1.5 with default parameters, installed on the Yale High Performance Computing Cluster. Cluster generation and error rates were also evaluated using the pipeline. We next aligned reads to the mouse genome, *Mus musculus* MM9, using Bowtie [48] as implemented in TopHat [49]. The splice junctions were mapped by TopHat by using the pre-built splice junction library provided by the authors. We used Cufflinks [50] to assemble transcripts using RefSeq as the reference list.

## Results

### Expression of CCK receptors in mouse brain is developmentally regulated

**Fig 1:**
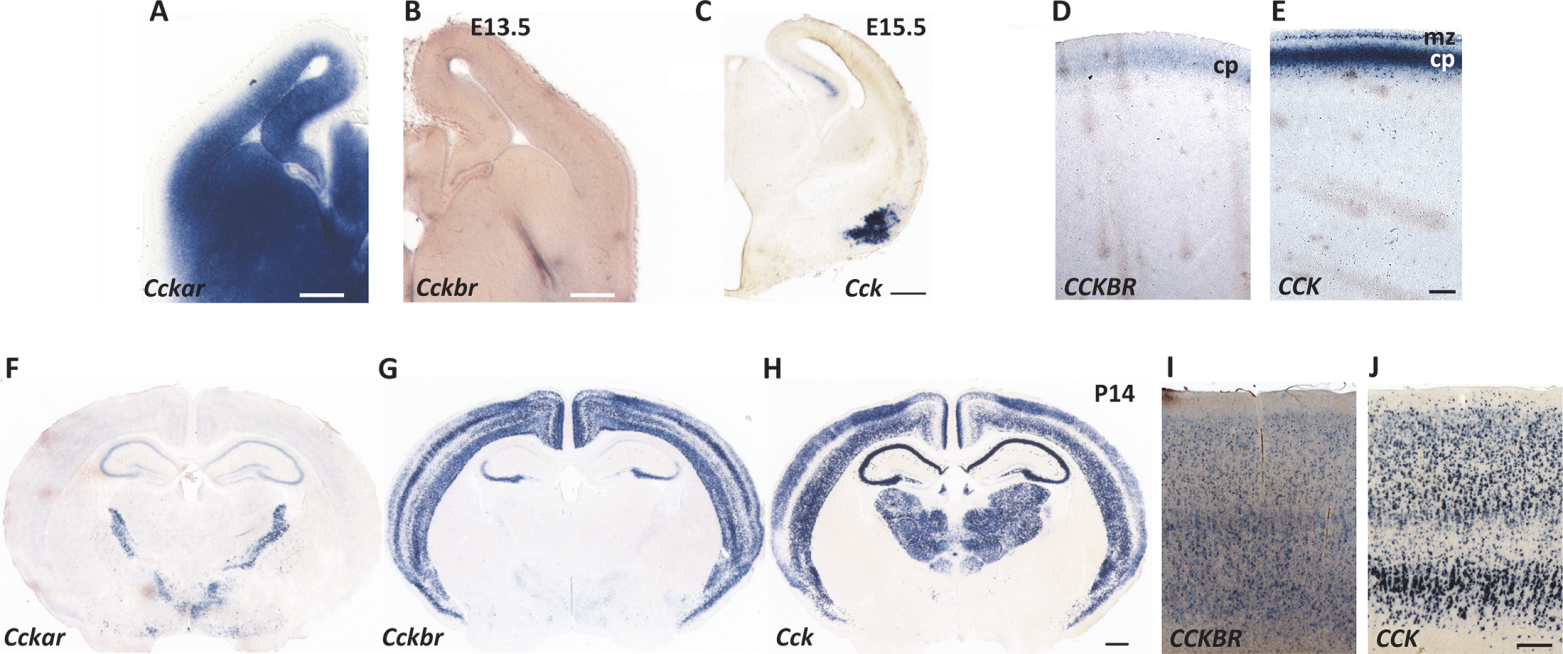
Expression of CCK and its receptors in mouse and human brain. (A, B) *Cckar* is highly expressed in the developing mouse brain at E13.5, contrary to *Cckbr* mRNA, which is barely detectable at this stage. Scale bar, 200 μm. (C) Select embryonic cells express *Cck* at E15.5. Scale bar, 500 μm (D and E) Expression of *CCKBR* (D) and *CCK* (E) in human fetal neocortex at 20 gestational weeks. Both transcripts are detected in the developing cortical plate (cp); *CCK* is also expressed in the marginal zone (mz). Scale bar, 0.5 mm. (F-H) By the second postnatal week, *Cckar* becomes restricted to the hippocampus and select extracortical sites, whereas *Cckbr* is highly expressed in the neocortex; *Cck* is also robustly expressed in the neocortex, hippocampus and thalamic nuclei. Scale bar, 100 μm. Note the opposite trends in expression levels of the two receptors at embryonic and postnatal stages. (I and J) Expression of *CCKBR* (I) and *CCK* (J) in human adult cortex (temporal lobe). Both transcripts are detected throughout all cortical layers with *CCK* being more robustly expressed. Scale bar, 0.5 mm.

The diverse physiological functions of CCK and its receptors in adult brain, prompted us to investigate whether they also played a role during development. We sought to determine the mRNA expression of the two receptors, as all previous studies of CCK receptor distribution were based on binding assays [13–18]. Contrary to its being considered the peripheral receptor, we discovered robust and widespread expression of *Cckar* mRNA in the developing brain by in situ hybridization from at least embryonic day (E) 13.5 onward, whereas *Cckbr* transcripts were not detected until late embryonic stages (Fig 1A and 1B). Confirming these observations, transcriptome analysis of normal mouse neocortex at two different embryonic stages (E13.5 and E17.5) demonstrated opposite trends in expression levels of *Cckar* (high, early vs. low, late) and *Cckbr* (low, early vs. high, late) during embryogenesis (our unpublished data). *Cck* is expressed in select embryonic cell populations (Fig 1C), previously shown to include tangentially migrating interneuron precursors [51]. By the second postnatal week, however, *Cckbr* is robustly upregulated in the neocortex, as is *Cck*, whereas *Cckar* is downregulated in the neocortex and becomes restricted to the hippocampus and select extra-cortical areas (Fig 1F–1H). In contrast, in human fetal brain (20 gestational weeks), we detected expression of *CCKBR* as well as *CCK*, but not of *CCKAR* (Fig 1D and 1E), extending to human previous reports of species-specific differences in receptor expression. *CCKBR* and *CCK* (but, again, not *CCKAR*) were also expressed in human adult frontal and temporal cortices (Fig 1I and 1J). Analyses of *Cckar* and *Cckbr* expression trajectories across neocortical layers of postnatal mouse brain over time ([52] and http://hbatlas.org/mouseNCXtranscriptome/) and of *CCKAR* and *CCKBR* exon array signal intensity in human brain across development and adulthood ([53–55] and http://hbatlas.org/) were both in agreement with our experimental findings (the corresponding graphs can be retrieved using the web links above and are also shown in S1 Fig).

These observations establish that the two CCK receptors are dynamically expressed, displaying *distinct* and nearly mutually exclusive patterns in embryonic and postnatal mouse brain, and are in agreement with previous reports that interneuron precursors and some cortical pyramidal neurons express *Cck* [21, 22, 51]. Considered together with the robust expression of *Cck* and the relatively mild or absent anatomical defects, respectively, of *Cckar* and *Cckbr* mutant brains, these findings also suggested a possible synergy in CCK receptor action in embryonic and early postnatal life, prompting us to analyze brain development of *Cckar/Cckbr* double mutant mice.

### Defects in cortical development and axonal connectivity in mice lacking CCKAR and CCKBR

**Fig 2:**
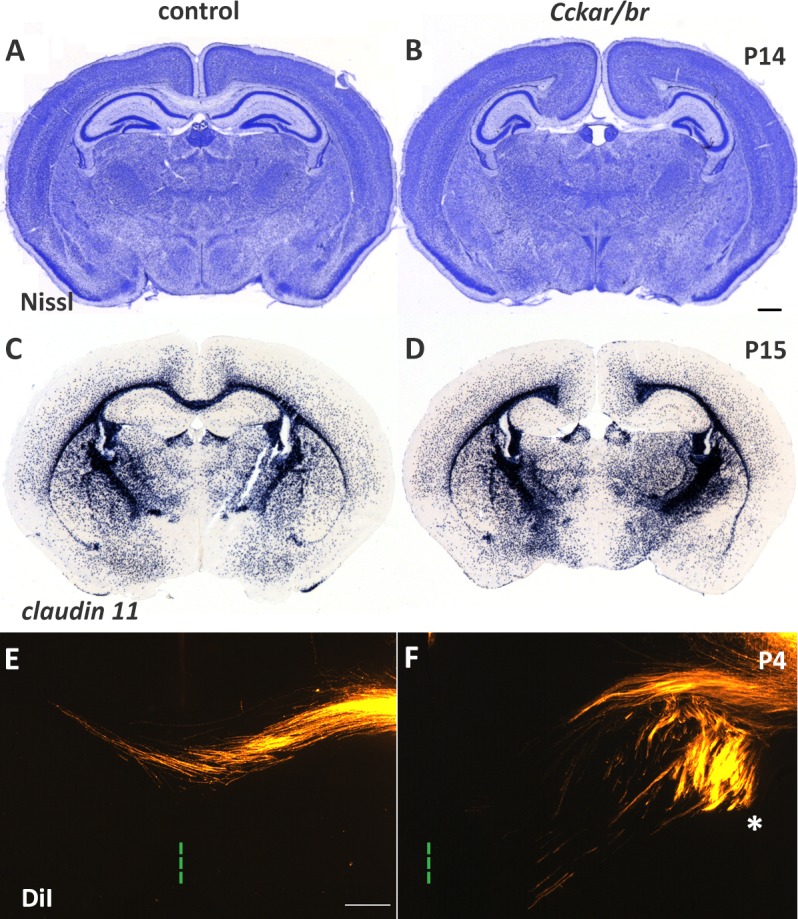
Defects in cortical development in *Cckar/br* mutant mice. (A and B) Nissl staining of coronal sections of control and *Cckar/br* mutant brains at postnatal day 14 (P14) reveals thickening of the cingulate cortex, agenesis of the corpus callosum, and lateral displacement of the hippocampus. (C and D) In situ hybridization with *claudin 11*, which is expressed in myelinated oligodentrocytes thus highlighting white matter tracts, shows that the commissural axons of the corpus callosum do not cross to the contralateral hemisphere in *Cckar/br* mutants (D) compared to control (C). Of a total of 16 brains from *Cckar/br* mutants that were fully sectioned and analyzed for callosal development, 13 had complete and 3 had partial agenesis of the corpus callosum. Scale bar, 100 μm. (E and F) Labeling of callosal projections with DiI shows that colossal axons fail to cross the midline in *Cckar/br* double mutants (F). Green lines (in E,F) and asterisk (in F) indicate the midline and Probst bundles, respectively. Scale bar, 200 μm.

The *Cckar/Cckbr* double homozygous mutant mice (JAX 129-*Cckar* ^*tm1Kpn*^ *Cckbr* ^*tm1Kpn*^/J; henceforth *Cckar/br* mutants), are viable and fertile, and have no obvious physical abnormalities despite their lacking functional CCK receptors [17, 33]). To examine gross brain cytoarchitecture in *Cckar/br* mutant mice we analyzed cortical organization at late embryonic stages and up to weaning (P21), by Nissl staining, revealing a spectrum of structural abnormalities, including expansion and thickening of the cingulate/retrosplenial cortex and lateral displacement and compression of the hippocampus (Figs 2A,2B and S4 Fig). Despite the selective enlargement of the cingulate cortex, the ventral and lateral cortical regions retained normal appearance, without a mediolateral shift of sensory cortical areas. Nissl staining also revealed agenesis of the corpus callosum, the largest commissural tract in the vertebrate brain that coordinates information between the two cerebral hemispheres (Fig 2A and 2B). In situ hybridization with *claudin 11*, expressed in myelinated oligodendrocytes, including those in white matter tracts [56, 57], confirmed failure of callosal axons to cross (Fig 2C and 2D). However, other major forebrain tracts such as the anterior, ventral hippocampal, and posterior commissures appeared normal, suggesting that commissural tracts were not globally affected (S2 Fig).

**Fig 3:**
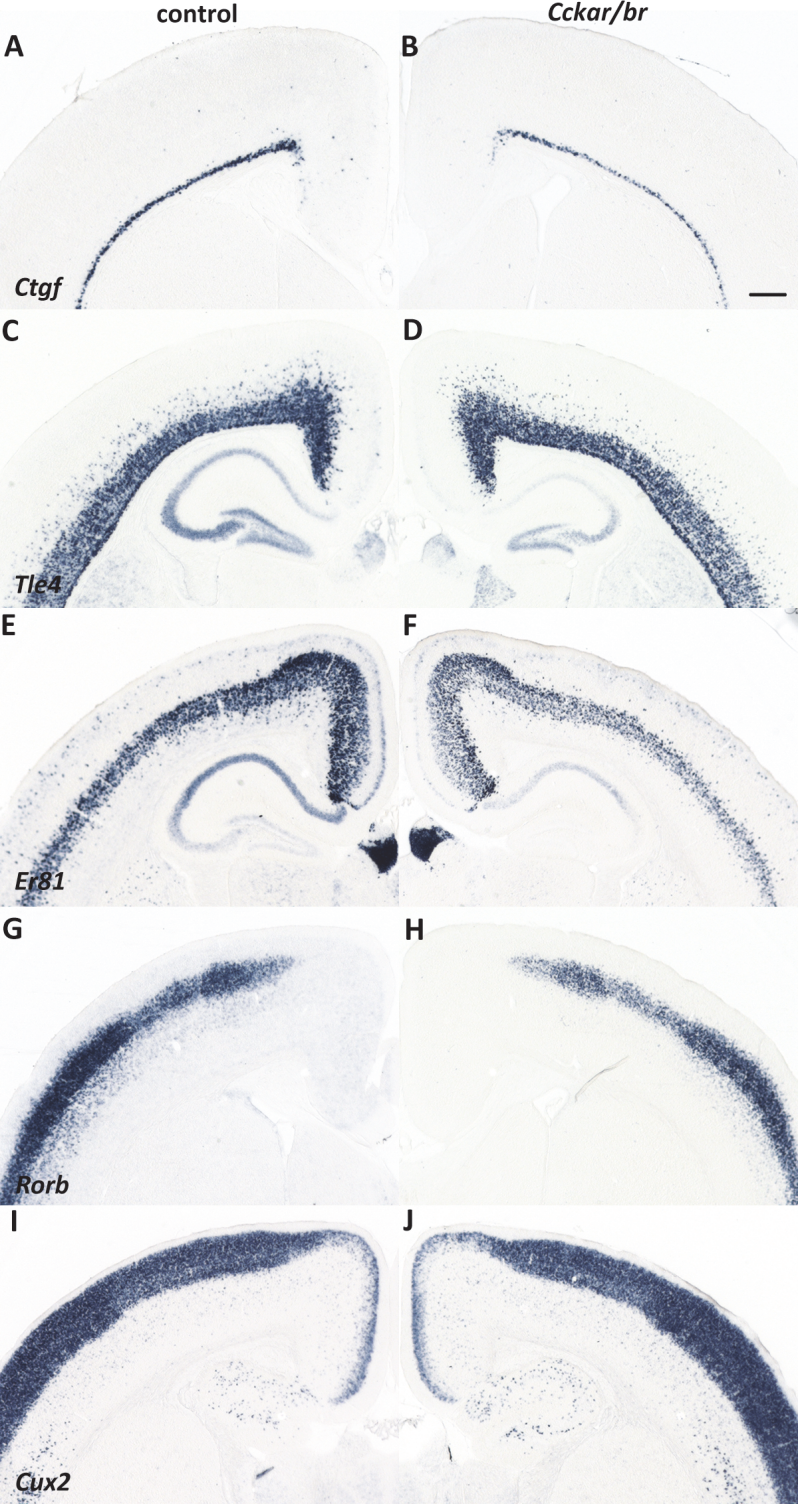
Normal cortical lamination in *Cckar/br* mutant mice. (A-J) In situ hybridization with cortical layer specific markers in control (A,C,E,G,I) and *Cckar/br* (B,D,F,H,J) littermates at P14. *Ctgf* (A,B): subplate; *Tle4* (C,D): L6, subplate; *Er81* (E,F): L5; *Rorb* (G,H): L4; *Cux2* (I,J): L2-4. Scale bar, 0.5 mm.

The differentiation and establishment of the corpus callosum depends on proper development of the dorsal midline, generation of callosal projection neurons (CPN) and their axons, axonal targeting and growth, and target localization and innervation in the contralateral hemisphere [58]. Agenesis of the corpus callosum is often associated with defects in CPN migration and/or specification [59–61], but can also result from alterations in cortical lamination. To rule out the latter possibility, we analyzed molecular markers of cortical layers (L) [including *Ctgf* (subplate); *Tle4* (L6, subplate); *Er81/Etv1* (L5); *Rorb* (L4); and *Cux2* (L2-4)] by in situ hybridization of representative sections of the entire neocortex, and concluded that layer formation and area patterning were grossly normal in *Cckar/br* mutants, however a possible reduction of a subset neocortical neuronal subtypes cannot be excluded at this point and warrants further investigation (Fig 3A–3J). In addition, in situ hybridization with *Tag1* (marker of commissural neurons) [62–64], *Satb2* (marker of CPN general identity) [59, 60] and *Lmo4* (expressed in CPN intermediate progenitors) [65, 66] between E14.5 and E17.5 revealed that CPN are generated in *Cckar/br* mutants (S3 Fig). Therefore, neither cortical layering nor failure of CPN generation appears to account for the agenesis of the corpus callosum in the *Cckar/br* mutants.

**Fig 4:**
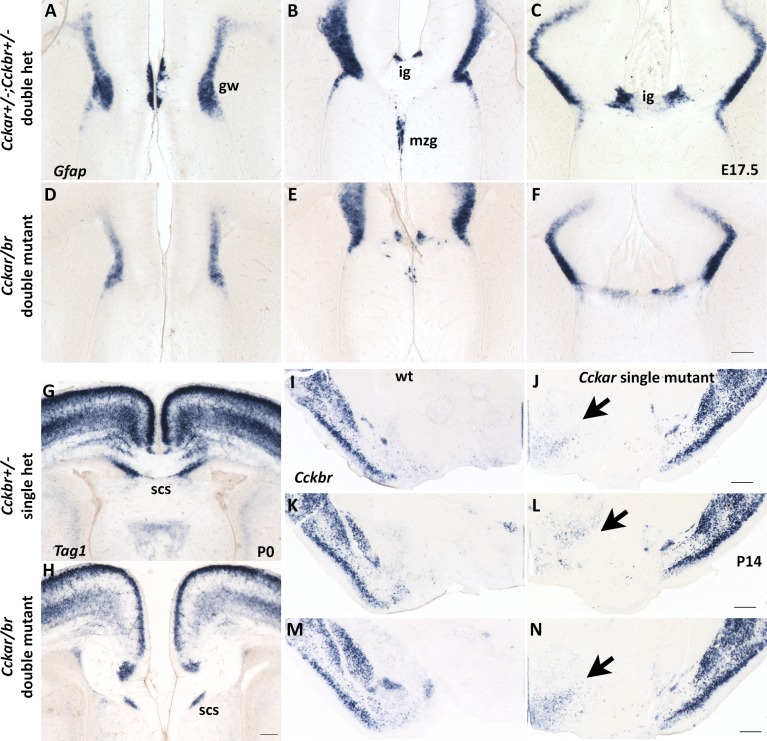
Abnormal differentiation of the midline in *Cckar/br* mutant embryos. (A-F) In situ hybridization with *Gfap*, a marker of midline glia at perinatal stages, reveals that two midline cell populations, the induseum griseum (IG) and midline zipper glia (mzg), are reduced in *Cckar/br* double mutants (D-F) compared even with *Cckar^+/-^;Cckbr^+/-^* double heterozygous embryos (A-C) at E17.5. Expression of *Gfap* at the glial wedge (gw) does not appear to be affected to the same extent, suggesting defective differentiation of the midline. Scale bar, 200 μm. (G and H) In situ hybridization with *Tag1*, a marker of commissural neurons, which also marks the subcallosal sling (scs), demonstrates that a continuous sling is present in control *Cckbr^+/-^* pups (G) at P0, but it fails to transverse the gap between the two hemispheres in *Cckar/br* mutants (H). Scale bar, 200 μm. (I-N) In situ hybridization demonstrates a modest increase in *Cckbr* transcripts in the ventral hypothalamus of *Cckar* single mutants (J,L,N) compared with wild type (I,K,M) at P14. The sections shown are at three different rostro-caudal levels. Scale bar, 0.5 mm.

Callosal axons originating in the neocortex represent the bulk of the corpus callosum, however, they depend on pioneering axons from the cingulate cortex, which pave the way by innervating the homotypic cingulate cortex in the contralateral hemisphere at E14-E15 [67–69]. Because the cingulate cortex is abnormal in the *Cckar/br* mutants, we examined medial cortical projections with DiI labeling at embryonic (E14.5 and E16.5) and early postnatal stages (P4) to view, respectively, the trajectory of pioneering and callosal axons. Pioneering axons were seen navigating towards the midline in both control and *Cckar/br* mutant embryos, suggesting that pioneering cingulate neurons were present; furthermore, callosal axons projected towards the midline, however, they failed to cross to the contralateral hemisphere in the mutants following instead aberrant trajectories and forming Probst bundles [70] on either side of the midline (Fig 2E and 2F; see also Figs 4H and 6D). Together, these observations suggest that although CPN neurons are generated, their axons fail to innervate the contralateral hemisphere, prompting us to investigate whether structural defects at the midline, including failure of midline fusion, might explain callosal agenesis in the *Cckar/br* mutants. We thus examined the differentiation of midline glia (glial wedge, midline zipper glia and induseum griseum), cell populations that have been implicated in callosal axon guidance [58, 71–73], as well as of the subcallosal sling (formerly known as “glial sling”), a transient structure that is comprised of migratory neurons [74, 75] and is necessary to bridge the gap between the two hemispheres providing a substrate onto which callosal axons grow towards the contralateral hemisphere.

Expression of *Gfap*, a marker of midline glia at perinatal stages, was significantly reduced specifically in the induseum griseum and the midline zipper glia at E17.5 and in neonatal (P0, P1) *Cckar/br* mutants, compared even with double heterozygous controls; expression at the glial wedge, was also reduced (Fig 4A–4F). These observations suggested abnormalities with midline differentiation. Examination of serial sections from an entire brain stained for *Gfap* expression indicated that in *Cckar/br* double mutants, migration of *Gfap-*expressing cells towards the midline was clearly reduced compared with controls, as were the size and rostrocaudal extent of the induseum griseum and midline zipper glia. Midline defects were also evident in neonatal brains by in situ hybridization with *F-spondin* [76] and *Tag-1* [62] markers of commissural neurons (Fig 4G and 4H). Moreover, the differentiation of the subcallosal sling, visualized by staining with NeuN, and also examined by morphology on Nissl preparations, was abnormal in the *Cckar/br* double mutants. Indeed, a continuous sling failed to transverse the gap between the two hemispheres, as also indicated by significantly reduced *Tag1* expression, marking the sling {Wolfer, 1994 #261}, in *Cckar/br* mutants at P0 (Fig 4G and 4H). These observations suggest that midline differentiation was abnormal in the *Cckar/br* mutants and that the delayed development/misspecification of the midline zipper glia and the induseum griseum, together with the failure of the subcallosal sling to bridge the gap between the two hemispheres are the likely causes of callosal agenesis.

Different guidance mechanisms are in place in the environment of the callosal trajectory [58] and several chemoattractant and chemorepellant cues are crucial for proper development of the corpus callosum [77–83]. We thus examined the expression of various guidance molecules to establish whether callosal axons were prevented from crossing owing to cell non-autonomous defects in the *Cckar/br* mutants. We did not detect differences in expression of the chemorepellant *Slit2* in glial wedge or the induseum griseum [78], or of multiple Eph receptors (*EphB1*, *EphB3*, *EphA4 or EphA5*) that regulate midline crossing of callosal fibers [84, 85], or *Sema3f* [82] (not shown). Taken together these observations suggest that abnormal patterning or differentiation of the midline may be the underlying cause of corpus callosum agenesis in the *Cckar/br* mutants.

### Defects in corpus callosum development in mice lacking CCKAR and CCKBR

To assess the relative contributions of each receptor to the formation of the corpus callosum, we examined *Cckar* and *Cckbr* single mutants, which we generated by crossing the *Cckar/br* mutants (maintained in the 129/J background) to 129/SvJ wild type mice in order to maintain the mutations in a uniform genetic background, thus eliminating genetic variability known to influence corpus callosum development as well as to control for sporadic occurrence of callosal agenesis in some strains [86, 87]. We analyzed brain sections through the entire anterior/posterior extent of the corpus callosum between birth and P21 by Nissl staining, and for each genotype, we scored brains displaying complete (failure of all callosal axons to cross the midline) or partial (at least part of the tract could be identified) agenesis. The *Cckar/br* mutants (n = 16) had either complete (n = 13) or partial (n = 3; in these, at least some callosal axons could be seen crossing anteriorly, but none did so at posterior levels) agenesis of the corpus callosum. On the other hand, almost all (9 out of 10) *Cckar* homozygous single mutants had normal corpus callosum with the remaining showing complete agenesis, whereas *Cckbr* homozygous single mutants (n = 9), as well as wild type 129/SvJ mice (n = 7), all had normal corpus callosum. The cytoarchitecture of the cingulate cortex and hippocampus was normal in both single mutants. These observations suggest functional synergy of CCKAR and CCKBR in cingulate cortex and corpus callosum development, possibly with a more important contribution of CCKAR, and, likely, functional compensation between the two receptors in the formation of the major forebrain commissural track. In support of this notion, we detected a modest but reproducible increase of *Cckbr* expression by in situ hybridization in the ventromedial hypothalamus of *Cckar* single mutants; any increase in the neocortex would be masked by the already robust *Cckbr* expression in this region (Fig 4I–4N; see also Fig 1G).

### Neuronal migration defects in *Cckar/br* mutants

In light of the synergistic action of the receptors and the migration defects of neuroendocrine neurons and of olfactory bulb interneurons previously reported in *Cckar* single mutants [43, 44, 46, 88], we reasoned that the *Cckar/br* double mutants would also have neuronal migration abnormalities. We noticed that the rostral migratory stream (RMS), observed on Nissl stained coronal and sagittal brain sections, had abnormal morphology and was severely thickened in the *Cckar/br* mutants (S4 Fig), as was the subventricular zone (SVZ) (S4 Fig). Quantitative analyses revealed that the volume of the RMS (P14: control, 0.356 +/- 0.024; *Cckar/br*, 1.41575 +/- 0.19876; n = 3; p = 0.02379 and P21: control, 0.37083 +/- 0.0404819; *Cckar/br*, 0.63075 +/- 0.11605; n = 3; p = 0.03807) was significantly increased in the *Cckar/br* mice compared with controls, suggesting that the rostral migration of neuroblasts was affected, consistent with previous findings of migration defects in adult olfactory bulb interneurons in *Cckar* single mutants.

**Fig 5:**
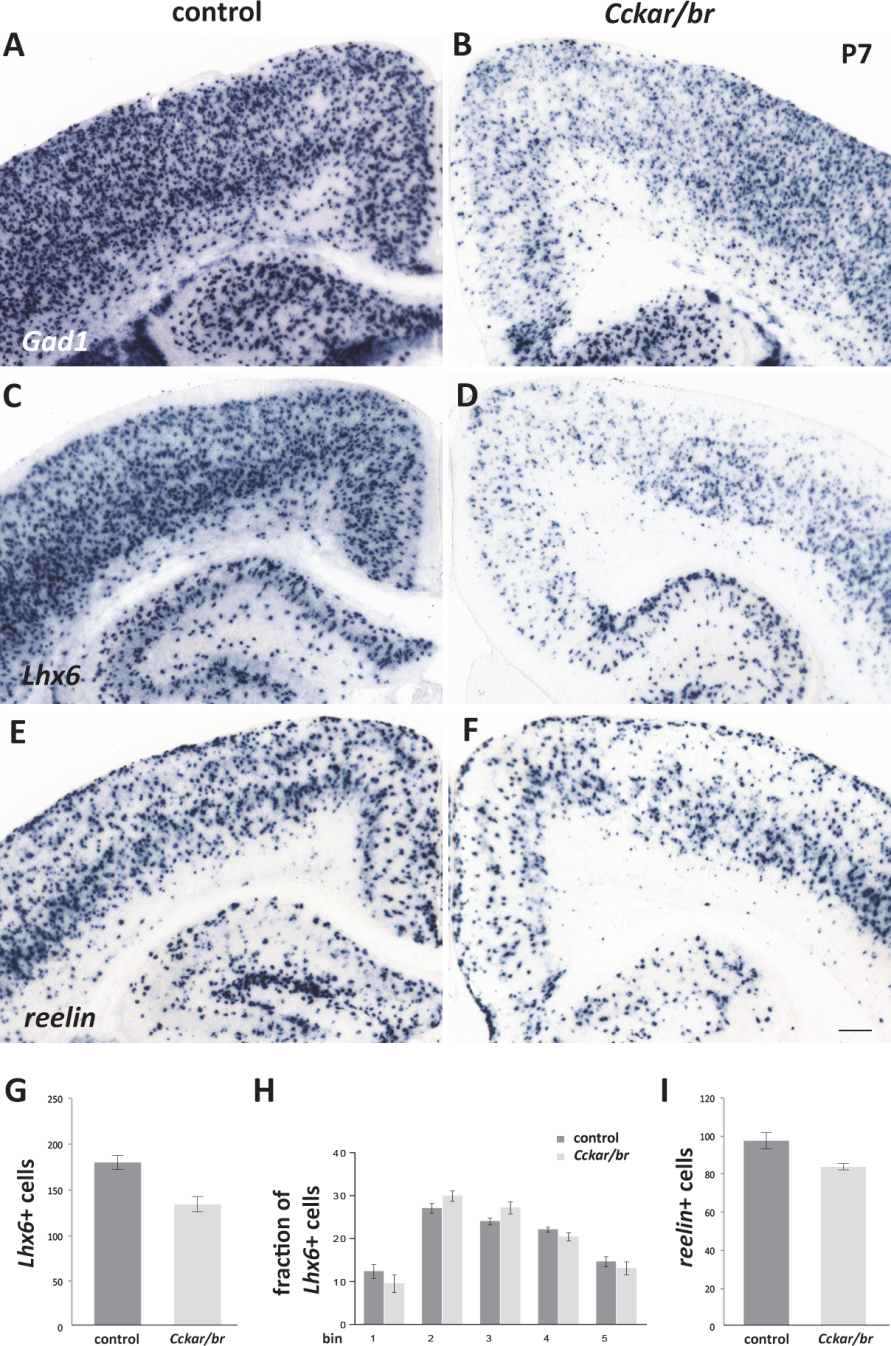
Neuronal migration defects in *Cckar/br* mutants. (A-F) In situ hybridization with markers of cortical interneurons at P7: *Gad1* (A,B) (a general interneuron marker); *Lhx6* (C,D) (a marker of MGE-derived interneurons); and *reelin* (E,F) (a marker of CGE- and pre-optic area derived interneurons) indicates a significant overall reduction of interneurons in *Cckar/br* mutants, irrespective of their origin. Scale bar, 200 μm. (G) Quantification of *Lhx6-*expressing interneurons. *Cckar/br* mutants have a 26% reduction in MGE-derived cortical interneurons at P7 (p = 0.0160, n = 3). (H) Distribution of *Lhx6-*expressing interneurons. Despite the reduction in their number, cortical interneurons are distributed in a similar fashion in control and *Cckar/br* mutant neocortex, suggesting that interneurons that successfully completed tangential migration are able to respond to local cues in the neocortex and settle into their final position. The fraction of *Lhx6-*positive interneurons per bin is similar in control and *Cckar/br* mutant neocortices (p is at least > 0.15 for all comparisons, n = 3). (I) Quantification of *reelin*-expressing interneurons. *Cckar/br* mutants have a 15% reduction in CGE- and POA-derived cortical interneurons at P7 (p = 0.0226, n = 4). Error bars represent s.e.m.

As shown in Fig 3, we did not detect any obvious defects in cortical lamination suggesting that migration of cortical pyramidal neurons was grossly normal. In contrast, we observed a noticeable reduction of cortical interneurons, which are generated in the medial and caudal ganglionic eminences (respectively, MGE and CGE) of the embryonic ventral forebrain and migrate tangentially into the cortex, where they assume their final laminar positions by radial migration [89–91]. Analysis of *Gad1* (a.k.a. *Gad67*, a general interneuron marker encoding glutamate decarboxylase 1, an enzyme involved in GABA biosynthesis), *Lhx6* (a marker of MGE-derived interneuronal lineages [92, 93]) and *reelin* (a marker of CGE- and pre-optic area [POA]- derived interneurons [91, 94]) at P7 indicated a significant overall reduction in *Lhx6* ^*+*^ and *reelin* ^*+*^ cortical interneurons in the *Cckar/br* mutants (Fig 5A–5F). Quantification of *Lhx6*-expressing cells at P7 (n = 3) indicated a 26% reduction (control: 179.3±7.839; *Cckar/br*: 133.3±8.373; p = 0.0160) in MGE-derived cortical interneurons in the mutants compared with controls (Fig 5G). However, their laminar allocation was not grossly affected (Fig 5H), indicating that those interneurons reaching the neocortex, were indeed able to respond to local guiding cues controlling their final position. Furthermore, quantification of *reelin*-expressing cells at P7 (n = 4) indicated a 15% reduction (control: 98.75±4.289; *Cckar/br*: 84.75±1.652; p = 0.0226) in CGE- and POA-derived interneurons in the *Cckar/br* mutants, compared to controls (Fig 5I). On the other hand, striatal cholinergic interneurons, which are born in the basal forebrain but do not migrate into the cortex, were generated in *Cckar/br* mutants in numbers comparable to wild type, as indicated by quantification of *Lhx8*-expressing cholinergic interneurons [95, 96] in a defined area of the striatum at P0 (control: 159.3±7.78; *Cckar/br*: 168.1±12.51, p = 0.55971) and at P7 (control: 93.9±5.55; *Cckar/*br: 105.2±9.66, p = 0.3238) (S5 Fig).

**Fig 6:**
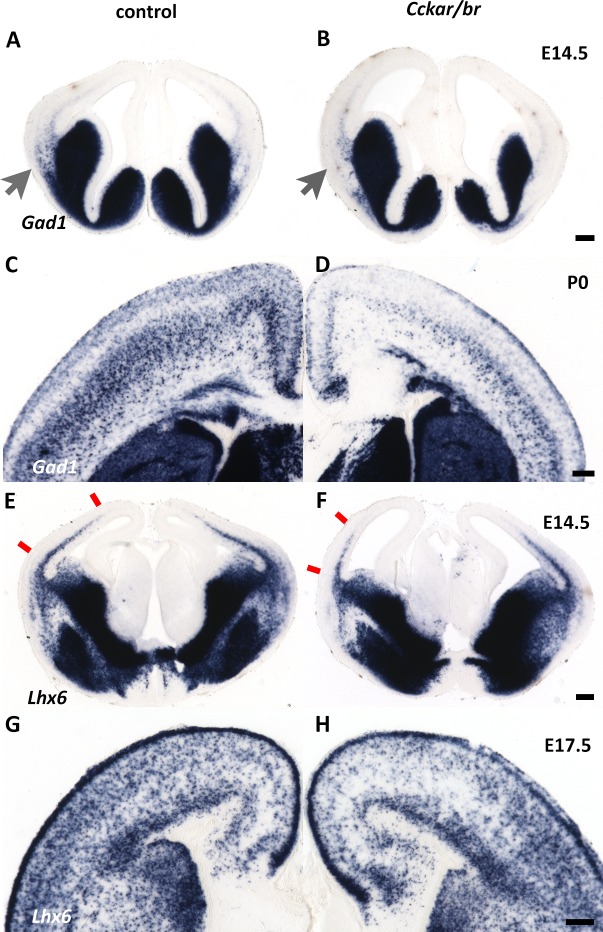
Defects in tangentially migrating interneurons in *Cckar/br* mutants. (A,B,E,F) Interneuron progenitors are generated normally in *Cckar/br* mutant embryos compared to controls, as demonstrated by in situ hybridization with *Gad1* (A,B) and *Lhx6* (E,F). However their tangential migration appears delayed in the mutants. Arrows in (A,B) indicate the onset of tangential routes employed by migrating interneurons. Note a delay/reduction in tangential migration in *Cckar/br* mutants. Red marks in (E,F) indicate the extent of tangential migration (deep route) in the neocortex. Scale bar (B,D), 200 μm. (C,D,G,H) In situ hybridization with *Gad1* and *Lhx6* at birth (P0, C,D) or late embryogenesis (E17.5, G,H) demonstrates that despite the delay in migration, a majority of interneurons settle into the neocortex. Scale bar (F,H), 200 μm.

A reduction in cortical interneuron number could be due to cellular malfunctions at the site of origin (e.g. fewer interneuron progenitors in the MGE/CGE), abnormal tangential migration, or failure to survive within the cortex. We examined the *Cckar/br* mutants at E14.5 (a day after the peak of interneuron progenitor generation in the MGE [97]), when tangential migration into the cortex is underway [98, 99]. In situ hybridization with *Gad1* and *Lhx6*, expressed at this stage by MGE-derived progenitors and post-mitotic migrating interneurons [89, 100], suggested that fewer interneurons were engaged in tangential migration (Fig 6A,6B,6E and 6F). Tangentially migrating interneurons employ two routes into the cortex, one superficial within the marginal zone and one deep at the subplate/SVZ interphase [101]. *Lhx6* and *Gad1* expression indicated that both routes were disrupted; the deep route appeared not as focused, and did not extend as far dorsally as in control littermates, and fewer migrating interneurons employed the superficial route into the cortex at this stage. Despite this migration delay, interneurons did reach the neocortex at later stages (Fig 6C,6D,6G and 6H), albeit in lower than normal numbers (Fig 5G and 5I).

Our findings suggest that tangential migration of interneurons into the cortex is partially disrupted in the *Cckar/br* mutants. For tangential migration to occur, different classes of interneurons respond to a variety of molecular cues via guidance receptors, the expression of which is under region-specific transcriptional control [89, 99]. Neuregulin-1, a short- and long-range chemoattractant, at E17.5 is expressed in the cortex and migrating interneurons express its receptor ErbB4 [102, 103]. Migrating MGE-derived interneurons expressed *ErbB4* in both *Cckar/br* and controls at E15.5 and E17.5, and we failed to detect any differences in *Nrg1* expression in the cortex between control and *Cckar/br* double mutants at E15.5 or E17.5 (S6 Fig). These observations suggest the abnormal tangential migration we observed cannot be explained by disruption in ErbB4 expression. The molecular factors controlling the tangential migration of interneurons originating in the CGE and pre-optic area remain to be defined, preventing detailed examination.

### Comparative transcriptional profiling offers insight into the brain abnormalities of *Cckar/br* mutant mice

To gain mechanistic insight into CCK receptor signaling and determine gene targets associated with CCK action, we performed comparative transcriptome analysis of *Cckar/br* vs. wild type control neocortical tissue at E17.5. We reasoned that RNA-Seq at this stage would be potentially informative for both developmental processes that are disrupted in the mutants, as at E17.5, the formation of the corpus callosum as well as tangential migration of interneurons are still underway, the latter nearing completion. The analysis was performed on two sets of biological triplicates (RNA Integrity Number [RIN] for control samples: 9.7, 9.6, 9.7 and for *Cckar/br* samples: 9.8, 9.8, 9.7). After mapping the RNA-seq data, we used pathway analysis (http://www.ingenuity.com/) to determine which biological pathways were differentially modulated in normal and *Cckar/br* mutant neocortex. As would be expected in the absence of functional CCK receptors, CCK-mediated signaling was significantly downregulated [-log(p-value): 6.64E-01; ratio: 2.83E-02], as were the ERK/MAPK signaling pathway [4.74E-01, 1.96E-02], which is known to control cell proliferation and migration induced by CCKBR and the transcriptional regulation of gastrin-sensitive genes, but also protein translation induced by CCKAR in several cellular contexts [104], the CXCR4 signaling pathway [6.45–01, 2.37E-02], and the BMP signaling pathway [4.98E-01, 2.5E-02] (Table 1). Two additional pathways were strongly affected: glutamate receptor signaling [2.68E00, 7.25E-02] and axonal guidance signaling [2.39E00, 3.24E-02] (Table 1). Within these pathways, we identified a number of candidate genes whose expression was modulated in *Cckar/br* mutants compared with controls, that could, on the basis of their known roles in brain development, provide a potential mechanistic explanation for the phenotypes we observed (Table 2). We selected for further study and validation three transcripts, two of which were significantly downregulated in the *Cckar/br* mutants, *Cxcl12* (fold change: -2.104, p = 6.14E-08) and *Bmp7* (fold change: -2.092, p = 2.13E-03), and one, *Nrp2*, that was upregulated, (fold change: 1.510, p = 3.29E-04). We note that *Grin3a* and *Grin2b*, encoding subunits of the NMDA receptor were also found to be upregulated (respectively, 2.033, p = 5.97E-04 and 1.707, 8.71E-04), but were not experimentally validated (Table 2; see Discussion).

**Table 1:** Pathway analysis (Ingenuity.com) of RNA-Seq data.

Pathway	-log(p-value)	ratio
**Glutamate Receptor Signaling**	2.68+00	7.25E-02
**Axonal Guidance Signaling**	2.39E00	3.24E-02
**CCK/gastrin Signaling**	6.64E-01	2.83E-02
**CXCR4 Signaling**	6.45E-01	2.37E-02
**BMP Signaling Pathway**	4.98E-01	2.5E-02
**ERK/MAPK Signaling**	4.74E-01	1.96E-02

**Table 2:** Select transcripts identified by comparative transcriptional profiling.

Transcript	Fold change Down-regulated in *Cckar/br*	p-value
***Cxcl12***	-2.104	6.14E-08
***Bmp7***	-2.092	2.13E-03
	**Up-regulated in *Cckar/br***	
***Nrp2***	1.510	3.29E-04
***Grin3a***	2.033	5.97E-04
***Grin2b***	1.707	8.71E-04

**Fig 7:**
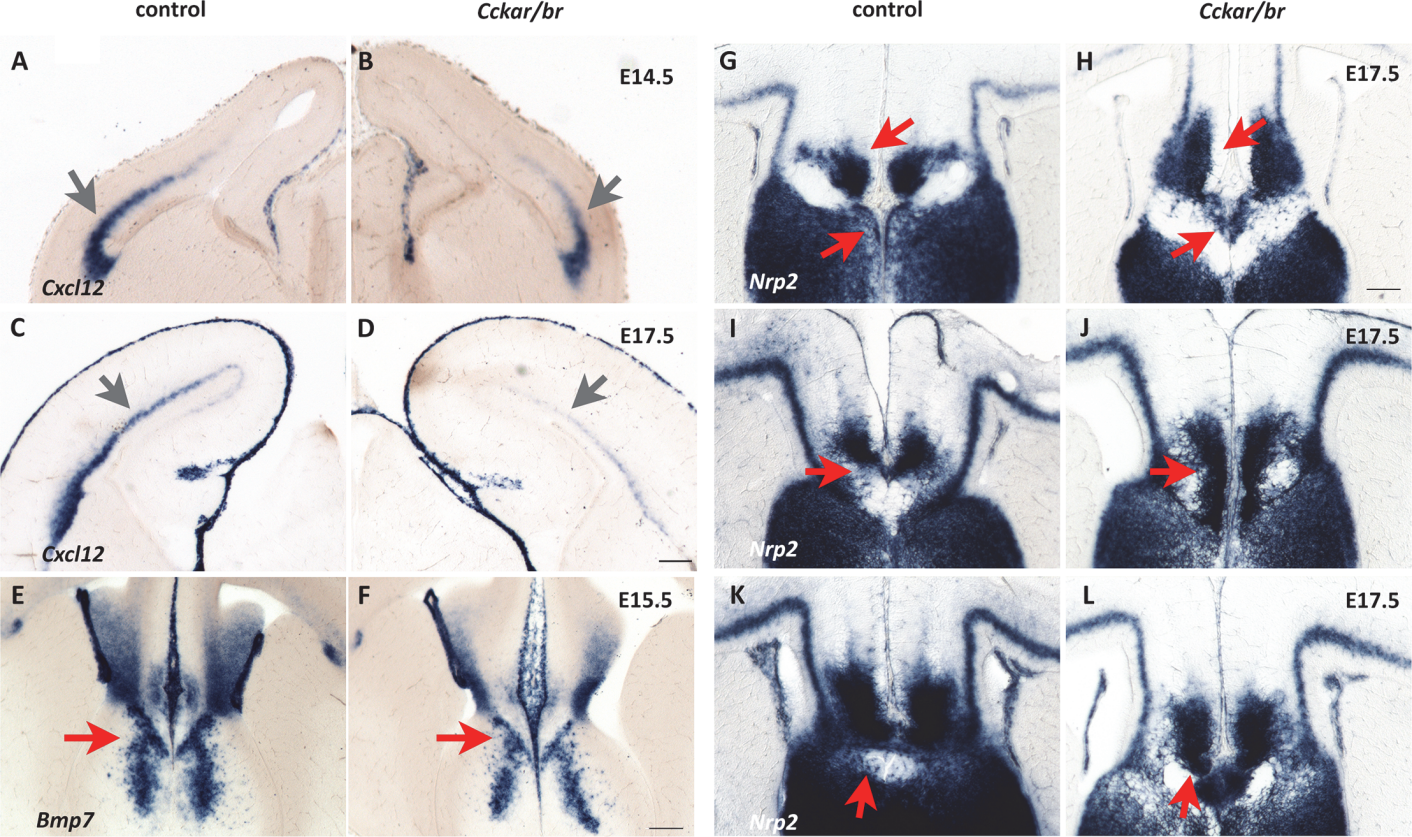
Validation of comparative transcriptional profiling findings. (A-D) *Cxcl12*, encoding a chemokine that is a known attractant for tangentially migrating interneurons, is downregulated (fold change: -2.104, p = 6.14E-08) in *Cckar/br* mutants. In situ hybridization demonstrates a reduction in *Cxcl12* transcripts in the SVZ (arrows) of mutant embryos compared to controls at E14.5 (A,B) and E17.5 (C,D). (E and F) *Bmp7*, encoding a secreted protein, is also downregulated (fold change: -2.092, p = 2.13E-03) in *Cckar/br* mutants. Arrows indicate lower expression levels of *Bmp7* in the midline of the mutants. Scale bar, 200 μm. (G-L) *Nrp2*, encoding the neuropilin 2 receptor, is upregulated (fold change: 1.510, p = 3.29E-04) in the *Cckar/br* mutants (H,J,L) compared to controls (G,I,K). The sections shown are at three different rostro-caudal levels (G and H, I and J, K and L). Arrows point to areas of differential expression levels of *Nrp2*, expanded in the mutants. Scale bar, 200 μm.

*Cxcl12* (previously called *Sdf1*) encodes a chemokine that is a known attractant for interneurons facilitating their migration through both the superficial and deep routes and their final laminar distribution [105–112]. CXCL12 is expressed by the meninges adjacent to the marginal zone (the superficial route), and by intermediate progenitor cells in the SVZ (the deep route). Indeed, in situ hybridization demonstrated that *Cxcl12* was expressed at lower levels in the SVZ of the *Cckar/br* mutants compared with double heterozygous animals; expression in the meninges was not affected (Fig 7A–7D).

*Bmp7*, the second transcript we investigated, encodes a secreted protein recently implicated in modulating callosal axon outgrowth via its participation in a signaling cascade from the meninges [113] as well as in the timely differentiation of midline “guidepost” cells involved in corpus callosum formation [114]. In *Cckar/br* mutants at E15.5 and E17.5, *Bmp7* was downregulated in the induseum griseum and the glial wedge (Fig 7E and 7F), providing a plausible explanation for the midline defects in the *Cckar/br* mutant mice.

Our comparative transcriptome analysis further indicated that *Nrp2 (Neuropilin 2)*, encoding a receptor for class III semaphorins, was more abundant in the *Cckar/br* mutants compared with controls. The neuropilin receptors, and their ligands, semaphorins, regulate repulsive axon guidance as well as neuronal cell migration [115]. Indeed, *Nrp2* was upregulated in the midline of *Cckar/br* mutant embryos at E14.5-E17.5, especially in the induseum griseum, whose orientation was further distorted (Fig 7G–7L). *Nrp2* is also expressed by tangentially migrating interneurons [99], and not surprisingly, we detected somewhat lower levels of expression of *Nrp2* in these cells, which are migrating in smaller than normal number in the *Cckar/br* mutants, again indicating defects at the palial-subpallial boundary and in the deep migratory route along the subplate/SVZ interphase (S7 Fig).

Thus, we were able to experimentally validate findings of RNA-Seq and gain insights into the potential mechanisms underlying the structural abnormalities documented in the *Cckar/br* mutant brains. These observations highlight the power of comparative transcriptome analysis of mutant vs. control tissues in understanding biological function.

## Discussion

Here we demonstrate that the CCK receptors are critical regulators of mammalian brain development. Constitutive loss of functional CCKAR and CCKBR, but not of either receptor alone, leads to midline structural anomalies in the developing brain resulting in agenesis of the corpus callosum and further impacts on tangential migration of cortical interneurons. These findings assign a new and previously unappreciated role to the CCK system in the development of the mammalian brain and suggest that the two receptors, whose expression is developmentally regulated and nearly complementary in embryonic and postnatal brain, display functional synergy and can compensate for one another.

The analysis of *Cckar/br* double mutant mice was prompted by our intriguing observations that one hand, CCKAR, long thought to be the peripheral receptor for CCK, is widely expressed in the embryonic mouse brain, as demonstrated by in situ hybridization and transcriptome analyses and on the other, that the expression of the two receptors is developmentally regulated with opposite trends, considered together with the relatively mild to even absent defects of each single mutant. The two receptors, which may be transiently co-expressed in some cells during late embryonic and early postnatal development, appear to act synergistically and to compensate for one another in certain contexts, e.g. in corpus callosum formation (this study) and in satiety control, as reported recently [116]. Contrary to the *Cckar/br* mutants, the *Cck* mutants apparently lack remarkable structural brain phenotypes (but do exhibit behavioral abnormalities related to satiation and control of food intake) {Lacourse, 1999 #41;Lo, 2008 #44;Hannibal, 2010 #235;Lo, 2012 #233}. *Cck* mutant mice do not upregulate gastrin (the peripheral ligand of the CCK receptors) in the brain, and *Cck/gastrin* double mutants have only been examined for (normal) pancreatic morphology {Lacourse, 1999 #41}. Despite these reports, however, whether brain development has been analyzed in any detail in the *Cck* mutants remains unclear. Should it be normal, however, the intriguing possibility of an additional ligand for these receptors in the brain should be investigated.

Our findings support and extend previous observations that correct establishment and patterning of the midline are essential for the formation of the corpus callosum [58, 61]. The lack of structural integrity of the midline, the distorted orientation and smaller size of the induseum griseum, and the reduction in size of other midline “guidepost” cell populations, including the subcallosal sling, considered together with the downregulation of *Bmp7* in the glial wedge and induseum griseum, suggest that abnormal differentiation of the midline is the most likely cause of callosal agenesis. BMP7 has indeed been implicated in callosal development {Sanchez-Camacho, 2011 #164}{Choe, 2012 #258}: genetic manipulation of BMP7 levels specifically in the meninges, normally expressing and secreting this molecule, either prevents callosal axons from crossing (when in excess), or leads to a larger corpus callosum (when reduced). BMP7 is also required for the proper differentiation of midline glia; in *Bmp7* null mutants these are defective, and the corpus callosum does not form. Considered together with our findings, these observations suggest a fundamental role of BMP7 in the formation of the corpus callosum. Our finding that other forebrain commissures are not disrupted in *Cckar/br* mice also suggests that cell non-autonomous mechanisms (such as the delayed development of the midline), rather than cell intrinsic defects of commissural neurons, are responsible for callosal agenesis. Thus, the *Cckar/br* mutants must now be added to the list of several mouse models with midline defects that have callosal phenotypes of varying penetrance [61]. The CCK receptors should also be evaluated as candidates for human syndromes of callosal agenesis for which a causative gene has not been identified.

Our findings also demonstrate neuronal migration abnormalities in the *Cckar/br* mutant mice. Previous analyses of *Cckar* single mutants indicated defects in two cell populations with opposing migratory routes: migration of neuroendocrine neurons that secrete gonadotrophin-releasing hormone (GnRH) [43] and fewer neuroblasts giving rise to adult olfactory bulb interneurons [44, 46]. GnRH-secreting neurons originate in the nasal compartment and migrate in association with the olfactory nerve to enter into the forebrain and reach their final destinations in the hypothalamus [117, 118]. Conversely, olfactory bulb interneurons originate in the adult SVZ and migrate via the RMS to the olfactory bulb, where they integrate into its circuitry [119]. The increase in thickness of the RMS suggests that olfactory bulb interneuron migration is affected in the *Cckar/br* mutants. Previous findings of concomitant impairment of corpus callosum formation and neuroblast migration from the SVZ to the olfactory bulb in *Cdk5* conditional mutant mice led to the suggestion that agenesis of the corpus callosum may interfere with the environment of the SVZ, thus contributing to the neuroblast migration defects [120], a hypothesis that also seems plausible for the *Cckar/br* mutants analyzed in this study.

We further observed that tangential migration of interneurons from their birthplace in subcortical areas towards the neocortex was delayed in the *Cckar/br* double mutants, and, consequently the number of neocortical interneurons was significantly reduced. The consequences of CCK receptor loss appear to be more pronounced in the subpopulation of interneurons migrating along the superficial route. MGE- and CGE- and POA-derived interneurons (respectively characterized by *Lhx6* and *reelin* expression) appear to be affected, suggesting that the defect is not specific to CCK-expressing neocortical interneurons, which are thought to derive from the CGE [28, 121, 122]. Although the molecular mechanisms guiding tangential migration of CGE-derived interneurons remain to be elucidated [91], we propose that the lower levels of *Cxcl12* expression that we detected in SVZ progenitors contribute to rendering the *Cckar/br* neocortex less attractive to tangentially migrating interneurons, regardless of their origin. Interestingly has been reported that the migration of GnRH neurons to the ventral forebrain, which is modulated by CCK and defective in *Cckar* single mutants [43], requires CXCL12/CXCR4 signaling [123]. Therefore, the CXCL12/CXCR4 signaling perturbations suggested by our study may underlie defects in several migrations over a distance in the *Cckar/br* mutants, including that of GnRH neurons [43] and cortical interneurons (this study).

Considered together, these defects not only highlight the important role of CCK-mediated signaling in the brain, but also have intriguing parallels. In the absence of functional CCK receptors, commissural axons fail to cross the midline to form the corpus callosum (this study); fewer cortical interneurons that have to travel across the pallial-subpallial boundary, enter the neocortex (this study) and fewer olfactory bulb interneurons reach their targets [44, 46, 88]; yet, more peripherally generated neuroendocrine neurons cross into the forebrain [43]. It appears therefore that CCK signaling is involved in regulating midline crossing by commissural axons and migratory travel across boundaries, two processes that have indeed been proposed to be analogous [91].

Comparative trasncriptome analysis led to the identification of two significantly downregulated transcripts in the *Cckar/br* mutant neocortex, encoding the chemokine CXCL12, a known attractant for tangentially migrating interneurons [106, 108–111], and the secreted factor BMP7, which was recently implicated in corpus callosum formation [113, 114]. Both were validated in vivo, and their modulation helps explain two of the phenotypes we detected in the *Cckar/br* mutant mice. A third transcript, *Nrp2*, which we also validated in vivo, is upregulated in the midline of the mutants, where it could be acting as a repellant to guide cingulate pioneer axons [82]. Interestingly, Nrp2, acting as an attractant, has also been implicated in the migration of GnRH neurons [124], and therefore, *Nrp2* upregulation may explain the increase in GnRH neurons in the *Cckar* mutants [43]. Finally, *Grin3a* and *Grin2b*, encoding subunits of the NMDA receptor, were upregulated in the C*ckar/br* mutants (this observations was not validated experimentally). Both are “juvenile” NMDA receptor subtypes that are developmentally replaced with “mature” subtypes during postnatal periods of activity-dependent rearrangement of synaptic connectivity, a developmental switch of functional importance [125–129]. An interplay of CCK signaling and NMDA receptor has been reported in several contexts, including food intake and anxiety behavior [130–132]; furthermore, CGE-derived CCK interneurons have a distinct NMDA subunit composition [133]. For all these reasons, the modulation of expression levels of juvenile NMDA receptor subtypes suggested by comparative transcriptomics, warrants further investigation. Our findings thus underscore the power of deep transcriptional profiling as a novel and fertile genomic approach in the identification of downstream targets of CCK signals and the elucidation of the mechanisms underlying CCK receptor function.

In conclusion, we document an important and additive (synergistic?) role of the two CCK receptors in the formation of the corpus callosum and tangential migration of cortical interneurons, highlighting a developmental role for CCK receptor signaling in the mammalian brain.

## Supporting Information

S1 Fig Developmental expression trajectories of *Cckar* and *Cckbr* in mouse and human brain (data retrieved from Brain Atlas; http://hbatlas.org/mouseNCXtranscriptome/ and http://hbatlas.org/). Abbreviations: SgL (subgranular layer); L4 (layer 4); IgL (infragranular layer); NCX (neocortex); HIP (Hippocampus); AMY (amygdala); STR (striatum); MD (mediodorsal nucleus of the thalamus); CBC (cerebellar cortex).
(TIF)

S2 Fig Formation of anterior (ac), and posterior (pc) commissures. (TIF)

S3 Fig In situ hybridization with *Satb2* and *Lmo4*. (TIF)

S4 Fig Morphology of the rostral migratory stream (RMS) and subventricular zone (SVZ). (A,B) are sagittal sections (anterior to the left); (C-I) are coronal sections.
(TIF)

S5 Fig In situ hybridization with *Lhx8*. (TIF)

S6 Fig In situ hybridization with *Nrg1* and *ErbB4*. (TIF)

S7 Fig In situ hybridization with *Nrp2*. (TIF)

S1 Table Primers for in situ hybridization. (DOCX)
