# Supplementary figures and images for: Functional Synergy between Cholecystokinin Receptors CCKAR and CCKBR in Mammalian Brain Development
Source: PLoS One. 2015 Apr 15;10(4):e0124295. doi: 10.1371/journal.pone.0124295 (PMC4398320; doi:10.1371/journal.pone.0124295)

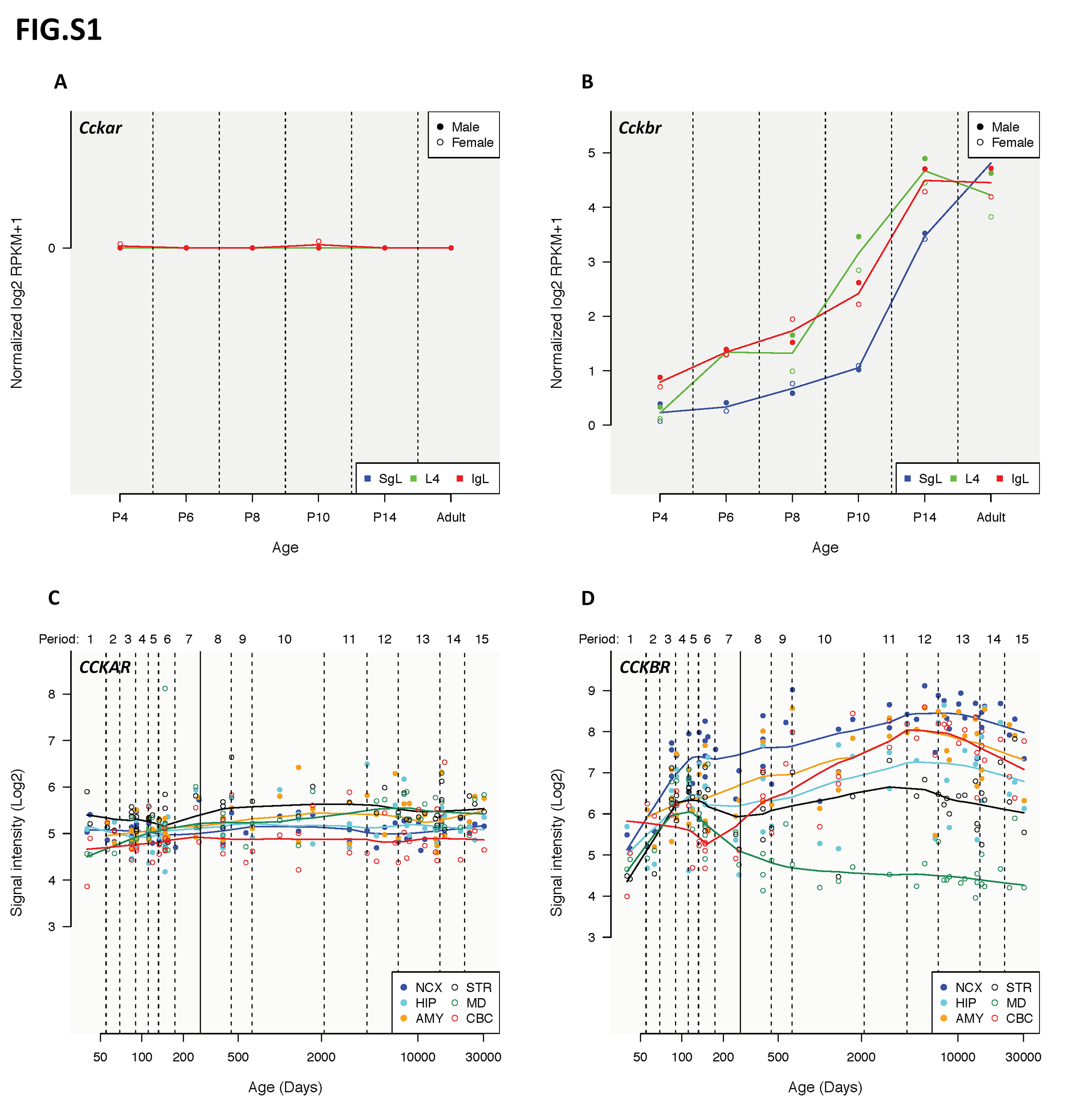

Supplement: S1 Fig — Abbreviations: SgL (subgranular layer); L4 (layer 4); IgL (infragranular layer); NCX (neocortex); HIP (Hippocampus); AMY (amygdala); STR (striatum); MD (mediodorsal nucleus of the thalamus); CBC (cerebellar cortex). (TIF) [file pone.0124295.s001.tif]

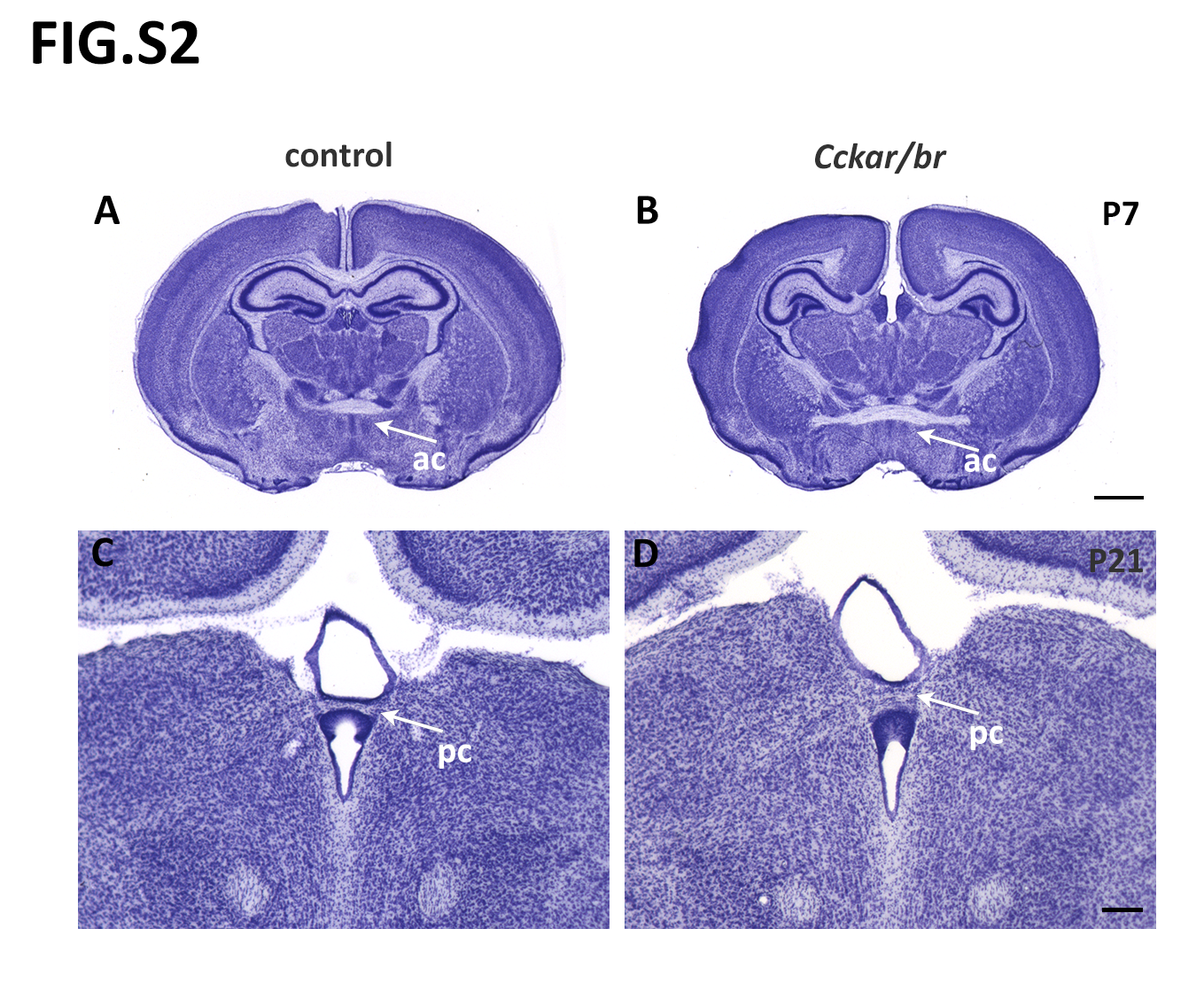

Supplement: S2 Fig — (TIF) [file pone.0124295.s002.tif]

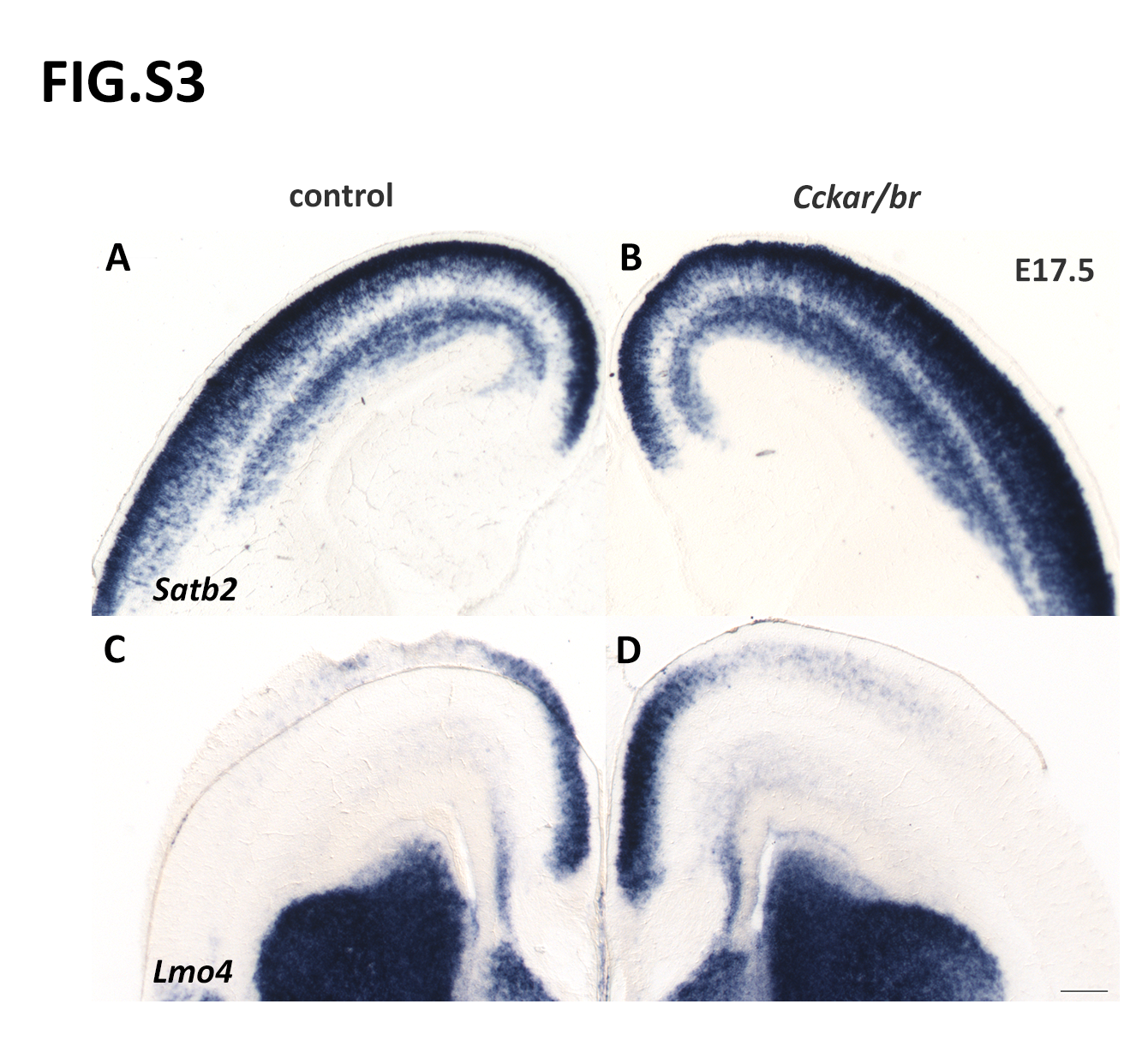

Supplement: S3 Fig — (TIF) [file pone.0124295.s003.tif]

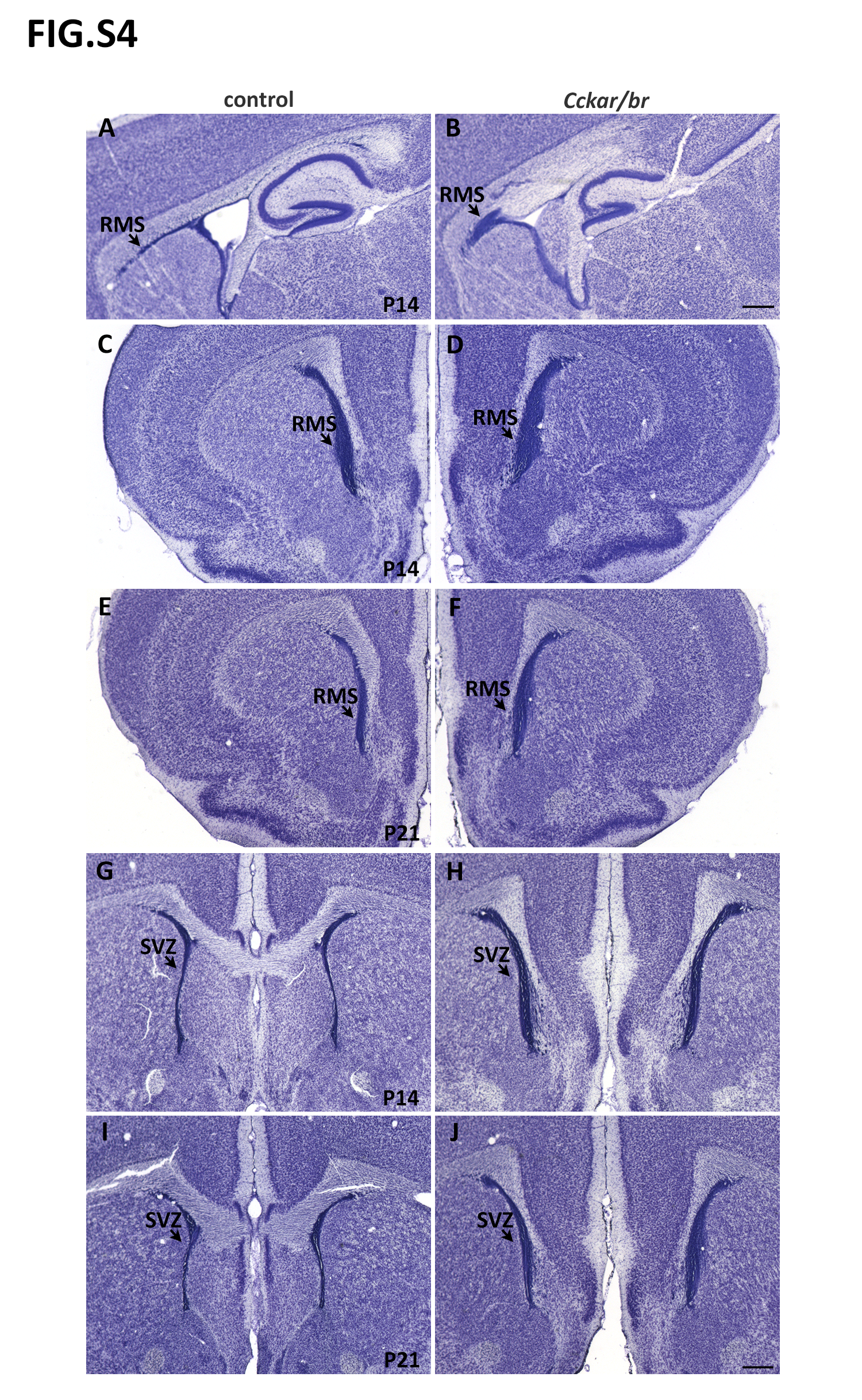

Supplement: S4 Fig — (A,B) are sagittal sections (anterior to the left); (C-I) are coronal sections. (TIF) [file pone.0124295.s004.tif]

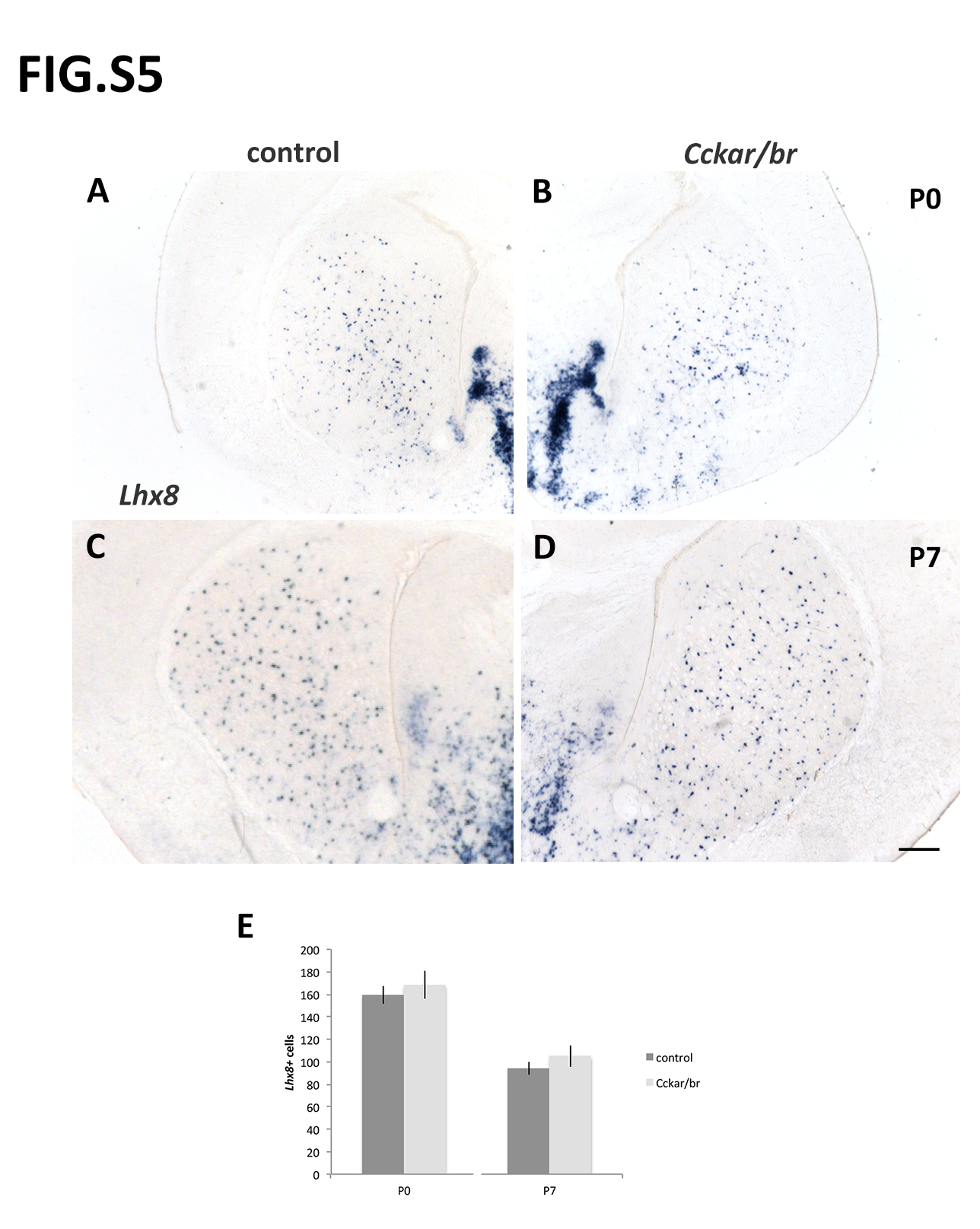

Supplement: S5 Fig — (TIF) [file pone.0124295.s005.tif]

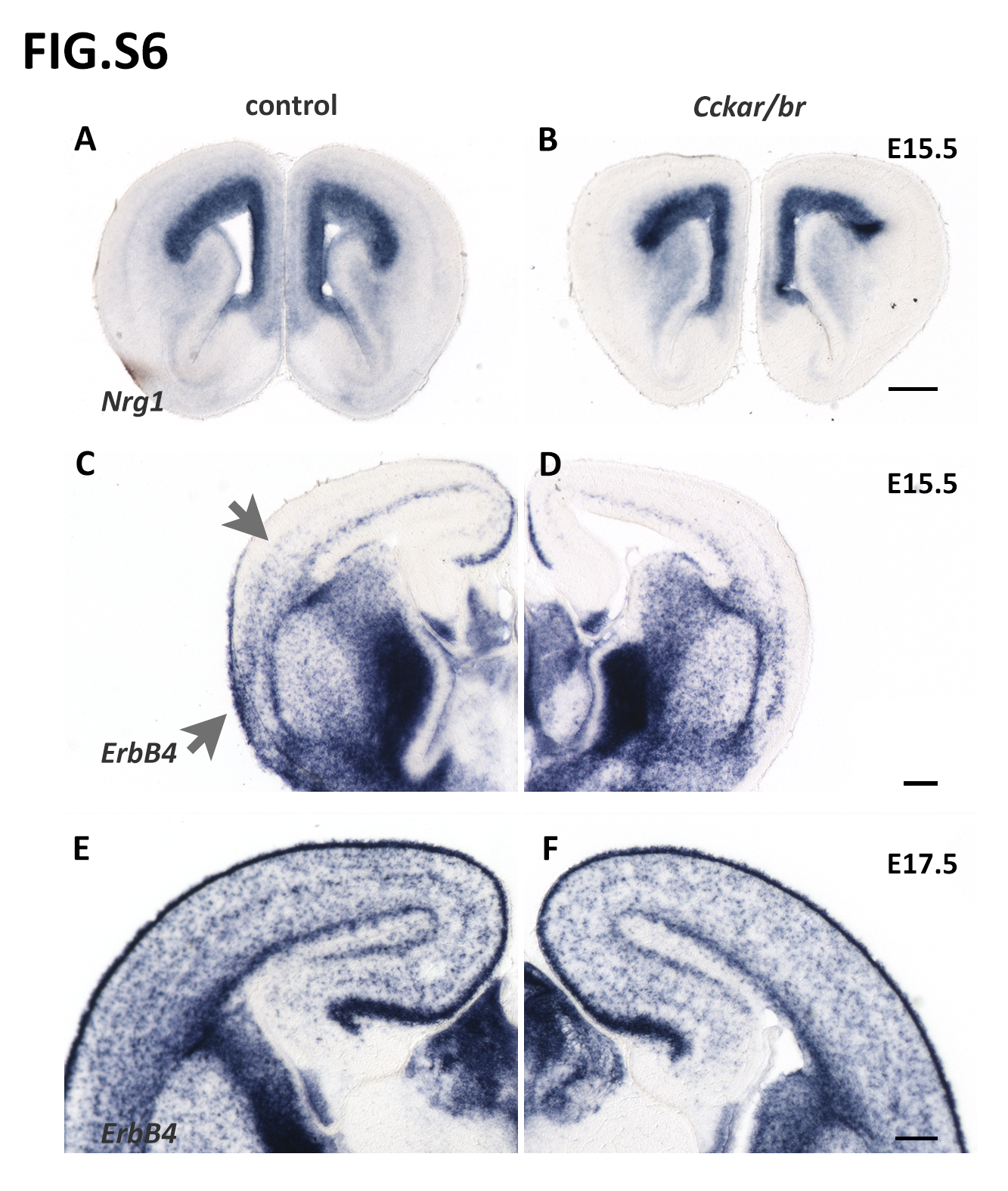

Supplement: S6 Fig — (TIF) [file pone.0124295.s006.tif]

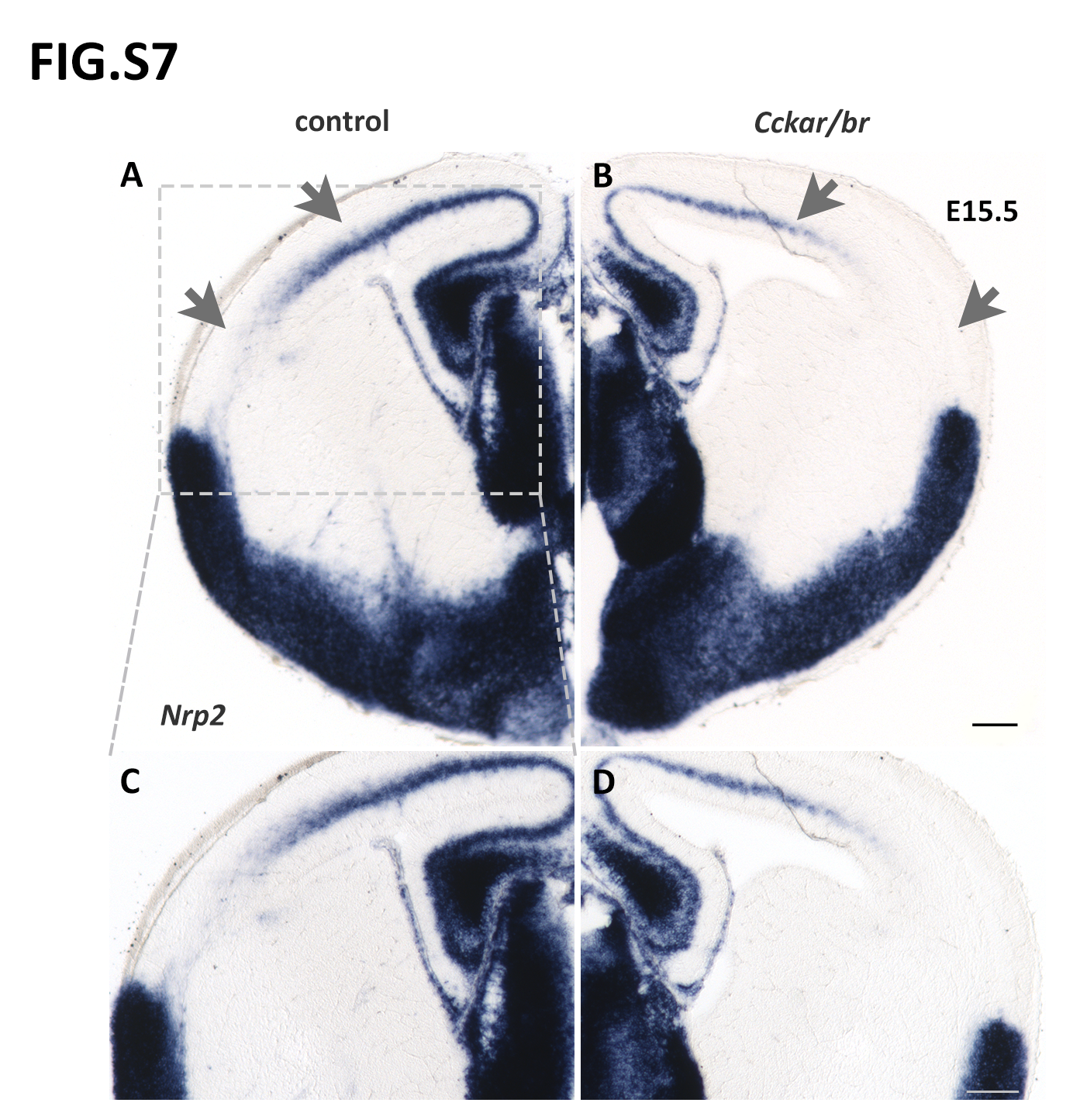

Supplement: S7 Fig — (TIF) [file pone.0124295.s007.tif]
