# Supplementary material for: Functional Synergy between Cholecystokinin Receptors CCKAR and CCKBR in Mammalian Brain Development
Source: PLoS One. 2015 Apr 15;10(4):e0124295. doi: 10.1371/journal.pone.0124295 (PMC4398320; doi:10.1371/journal.pone.0124295)
Supplement: S1 Table — (DOCX) [file pone.0124295.s008.docx]

**Table S1. Primers for in situ hybridization**

| Gene | Forward primer | Reverse primer |  |  |  |
| --- | --- | --- | --- | --- | --- |
| *Cckbr* | 5’-cgtgcttctgctaatactgctg-3’ | 5’-tgctatggaccgtatgtctgtc-3’ |  |  |  |
| *Cck* | 5’-gtatgtctgtgcgtggtgatg-3’ | 5’-agcatagcaacattaggtctgg-3’ |  |  |  |
| *Nrp2* | 5’-tccacacaatctggactgtacc-3’ | 5’-tgtcatagtgcatgttcccttc-3’ |  |  |  |
| *CCKAR* | 5’-tcttgtgaatggaagcaacatc-3’ | 5’-gtaggacaggaggaggatgaag-3’ |  |  |  |
| *CCKBR* | 5’-cctctgagcacgtgttactgc-3’ | 5’-caacattttcagaccaggagtc-3’ |  |  |  |
| *CCK* | 5’-cctctgagcacgtgttactgc-3’ | 5’-caacattttcagaccaggagtc-3’ |  |  |  |
